# Continuous monitoring of suspended sediment concentrations using image analytics and deriving inherent correlations by machine learning

**DOI:** 10.1038/s41598-020-64707-9

**Published:** 2020-05-22

**Authors:** Mohammad Ali Ghorbani, Rahman Khatibi, Vijay P. Singh, Ercan Kahya, Heikki Ruskeepää, Mandeep Kaur Saggi, Bellie Sivakumar, Sungwon Kim, Farzin Salmasi, Mahsa Hasanpour Kashani, Saeed Samadianfard, Mahmood Shahabi, Rasoul Jani

**Affiliations:** 1 Department of Water Engineering, University of Tabriz, Tabriz, Iran; 2 Department of Civil Engineering, Near East University, P.O. Box 99138, Nicosia, North Cyprus, Mersin 10 Turkey; 3 GTEV-ReX Limited, Swindon, UK; 4 Department of Biological and Agricultural Engineering & Zachry, Department of Civil Engineering, Texas A & M University, College Station, TX 77843-2117 USA; 5 Istanbul Technical University, Department of Civil Engineering, Istanbul, Turkey; 6 Department of Mathematics and Statistics, University of Turku, Turku, FIN-20014 Finland; 7 Department of Computer Science, Thapar Institute of Engineering and Technology, Patiala, India; 8 Department of Civil Engineering, Indian Institute of Technology Bombay, Powai, Mumbai 400076 India; 9 Department of Railroad Construction and Safety Engineering, Dongyang University, Yeongju, 36040 Republic of Korea; 10 Department of Water Engineering, Faculty of Agriculture and Natural Resources, University of Mohaghegh Ardabili, Ardabil, Iran; 11 Department of Civil Engineering, Tabriz Branch, Islamic Azad University, Tabriz, Iran

**Keywords:** Hydrology, Civil engineering

## Abstract

The barriers for the development of continuous monitoring of Suspended Sediment Concentration (SSC) in channels/rivers include costs and technological gaps but this paper shows that a solution is feasible by: (i) using readily available high-resolution images; (ii) transforming the images into image analytics to form a modelling dataset; and (iii) constructing predictive models by learning inherent correlation between observed SSC values and their image analytics. High-resolution images were taken of water containing a series of SSC values using an exploratory flume. Machine learning is processed by dividing the dataset into training and testing sets and the paper uses the following models: Generalized Linear Machine (GLM) and Distributed Random Forest (DRF). Results show that each model is capable of reliable predictions but the errors at higher SSC are not fully explained by modelling alone. Here we offer sufficient evidence for the feasibility of a continuous SSC monitoring capability in channels before the next phase of the study with the goal of producing practice guidelines.

## Introduction

Monitoring Suspended Sediment Concentration (SSC) in open channels is explored in this paper towards the goal of developing an innovative technique based on high-resolution imagery to train predictive models by using machine learning techniques. If the goal can be realised, the outcome would potentially meet the demand for SSC field measurements. Measurements of SSC data both in time and space are reviewed by the U.S. Bureau of Reclamation^1,2^. Existing measurement techniques are labour-intensive, time consuming and costly^3,4^, which also suffer from uncertainty. These techniques provide an important variable for design and management of open channel systems. The feasibility of *continuous monitoring of SSC using image processing* can be a potentially significant technique and if successful, some of existing barriers to continuous measurements can be removed to prepare the ground for the goal of producing practice guidance at the next phases.

The capability for monitoring SSC is based on photometric features of Red, Green, and Blue (RGB), where modern cheap high resolution cameras are capable of capturing subtle changes in tone and colour, both expressed in bits. RGB imaging tracks down the changes in the colour of river water through simple high-resolution images. The correlation between SSC and colour variations of RGB-based high-resolution images is explored for the prediction of SSC in the riverine environment. Image processing has already been investigated successfully for monitoring surface water velocity during flood events^5–7^. A relationship between SSC and water transparency levels was advocated by Oxford^8^ as early as 1976 but more recently the techniques have diversified and those using field measurement techniques are based on acoustic backscatter, laser diffraction, turbidity, as well as various variations of using images by employing acoustic, physical (e.g. density and electric properties) and optical principles. Rai and Kumar^9^ review the research in the past 3 decades for continuous measurement technologies with respect to measuring acoustics, laser diffraction, turbidity and pressure differences and other principles including image capturing techniques and note that the research in this field is still topical.

Researchers have already devised working methods to quantify water quality using images from digital cameras^10–12^. The techniques for fine sediments are outlined by Turley *et al*.^13^; Moirogiorgou *et al*.^14^ outline a scheme for correlating some of image properties with SSC using 6 samples taken from the real world; and Hoguane *et al*.^15^ investigate the measurement of mineral suspended sediment concentration using optics and images on the basis of capacities to absorb the blue spectrum. The difference between the current work and reported research works on using images for various aspects of continuous measurement of suspended sediment is in using the parameterisation of cheap high-resolution images alone for continuous monitoring of SSC.

**Figure 1:**
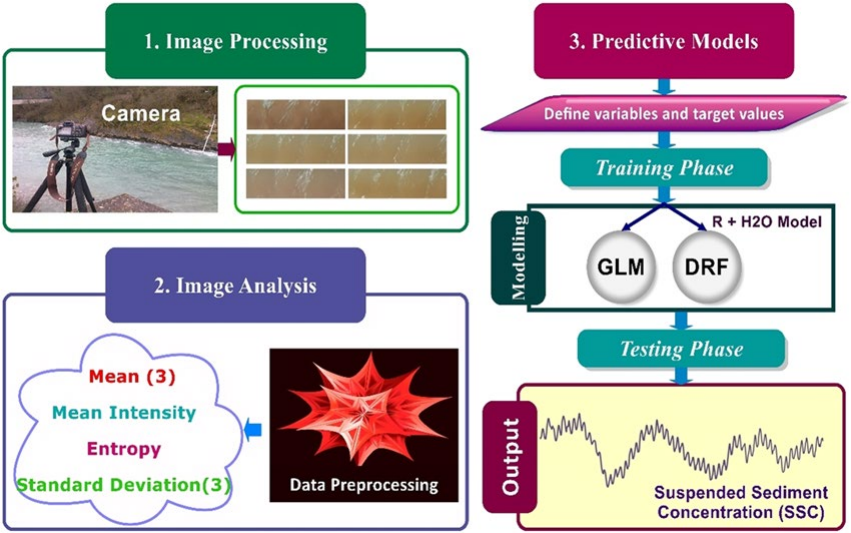
Prototype capability for continuous SSC monitoring in three steps.

The development of a capability is beyond the scope of a single research project but this often requires a lifecycle of activities, similar to the delivery of other tools and procedures. This paper takes only the preliminary step of the ‘proof-of-concept’ but other steps are outlined in due course. At this stage, it is necessary to consolidate the ideas for forming a set of procedures and to make a case for future works. The prototype capability, depicted in Fig. 1, requires the following activities: (i) setting up a laboratory flume for the generation of high-resolution imagery for SSC to test the idea; (ii) transforming the imagery into a set of parameters to serve as input data; and (iii) exploring existence of possible correlations within the input data. A review of each of these components is outlined below.

Image analytics connects the produced imagery to models, both of which are essential to produce input data to models. In this research, image analytics comprise 8 input variables, and comprise: Mean_3_, Mean intensity, Entropy, and Standard deviation_3_, (suffix ‘3’ corresponds to RGB); as well as target values in terms of measured SSC. If there is any connection in terms of inherent correlations within the image analytics, there is a good chance to be identified by artificial intelligence, machine learning models or similar techniques with the following features: (i) they are characteristically bottom-up techniques and therefore the models employ no prior theoretical or empirical laws but each model has its own strategy or heuristic rules and parameters; and (ii) they are data-driven, as the values of inherent parameters are learned from the data.

The paper uses the following techniques to learn any correlation in the image analytics: Generalized Linear Model (GLM) and Distributed Random Forest (DRF). The main focus of the paper is on a better understanding of continuous SSC monitoring but not on modelling, which serves the purpose of a tool. An overview of these techniques is as follows. **GLM** is used here as a regression technique, and the values of its inherent parameters are learnt by its estimator identified by maximising the log-likelihood, see Nelder and Wedderburn^16^ for more details. **DRF** is an ensemble learning technique, in which the performance of several weak learners is boosted via a voting scheme. It refers to a classifier that uses multiple trees to train and predict the samples^17^. These models are described later in the Methods section.

## Modelling results

### Datasets

The experimental data, described later in the Method, comprise 166 observations, which are divided randomly into 111 training datapoints and 55 testing datapoints (in the ratio of 2/3 and 1/3). Table 1 presents the statistical characteristics of measured SSC. The two models of GLM and DRF were constructed in the H2O platform through training and testing phases using the input data extracted from image analytics, as outlined in Fig. 1. The default parameters of each of these models are given later in Table 2. The experimental procedure is presented in the Methods section, where Figs. 5 and 6 illustrate the laboratory setup and gives examples of images from the test runs.

**Table 1:** Statistical characteristics of measured SSC.

	Variables	Datapoints	Mean	Variance	SD	Skewness	Maximum gr/lt	Minimum
Training Phase	SSC (gr/lt)	111	3.477	8.834	2.972	0.783	10	0.28
	Mean	3*111	0.466	0.010	0.100	−0.183	0.612	0.313
	Mean intensity	111	0.491	0.001	0.031	0.301	0.544	0.446
	Entropy	111	6.689	0.095	0.309	−0.184	7.260	5.852
	SD	3*111	0.052	0.0002	0.016	−0.013	0.092	0.023
Testing Phase	SSC (gr/lt)	55	3.506	8.901	2.983	0.772	9.9	0.32
	Mean	3*55	0.466	0.010	0.100	−0.182	0.610	0.306
	Mean intensity	55	0.492	0.0009	0.031	0.263	0.543	0.441
	Entropy	55	6.692	0.121	0.348	−0.841	7.346	5.723
	SD	3*55	0.052	0.0003	0.016	0.062	0.083	0.024

**Table 2:** Default model parameters.

GLM	DRF
Family = “gamma”;	mtries = 6
Lambda-search=TRUE	ntrees = 500
nlambdas = 100	max-depth = 20
Solver = “IRLSM”	nfolds = 5
Link = “inverse”	score_each_iteration = T
nfolds = 5	Estimated parameters: number of tree= 500
Estimated parameters: nlambda = 100	min depth=8 max depth=13
lambda max = 4.9555	
lambda min = 0.05191	

Total Number of datapoints: 166; Datapoints for training: 111; Datapoints for testing: 55; their ratio: 33%:67%.

**Figure 2:**
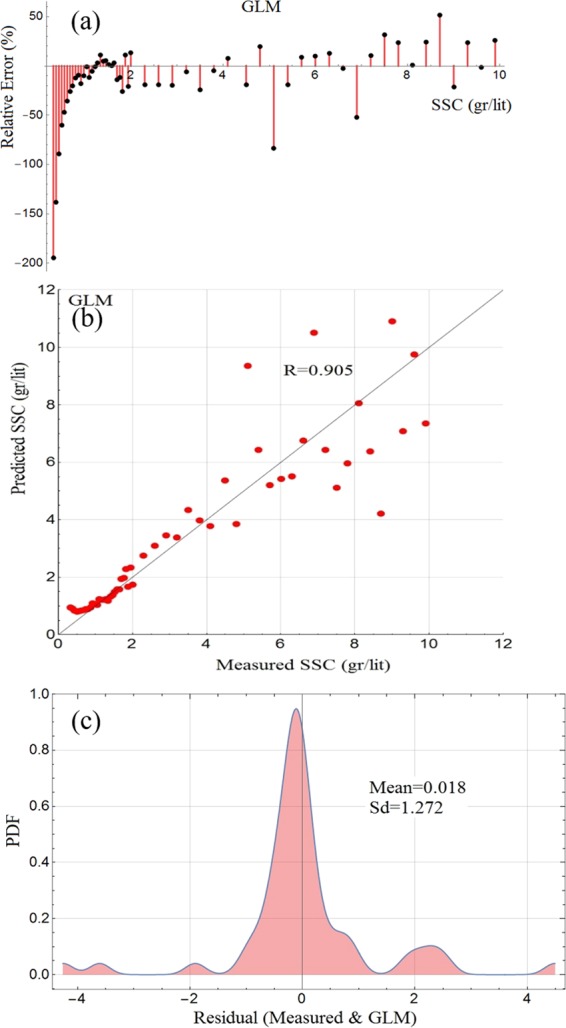
Results of GLM for the testing phases: (**a**) Relative error plot; (**b**) scatter diagram; (**c**) PDF plot of residuals.

**Figure 3:**
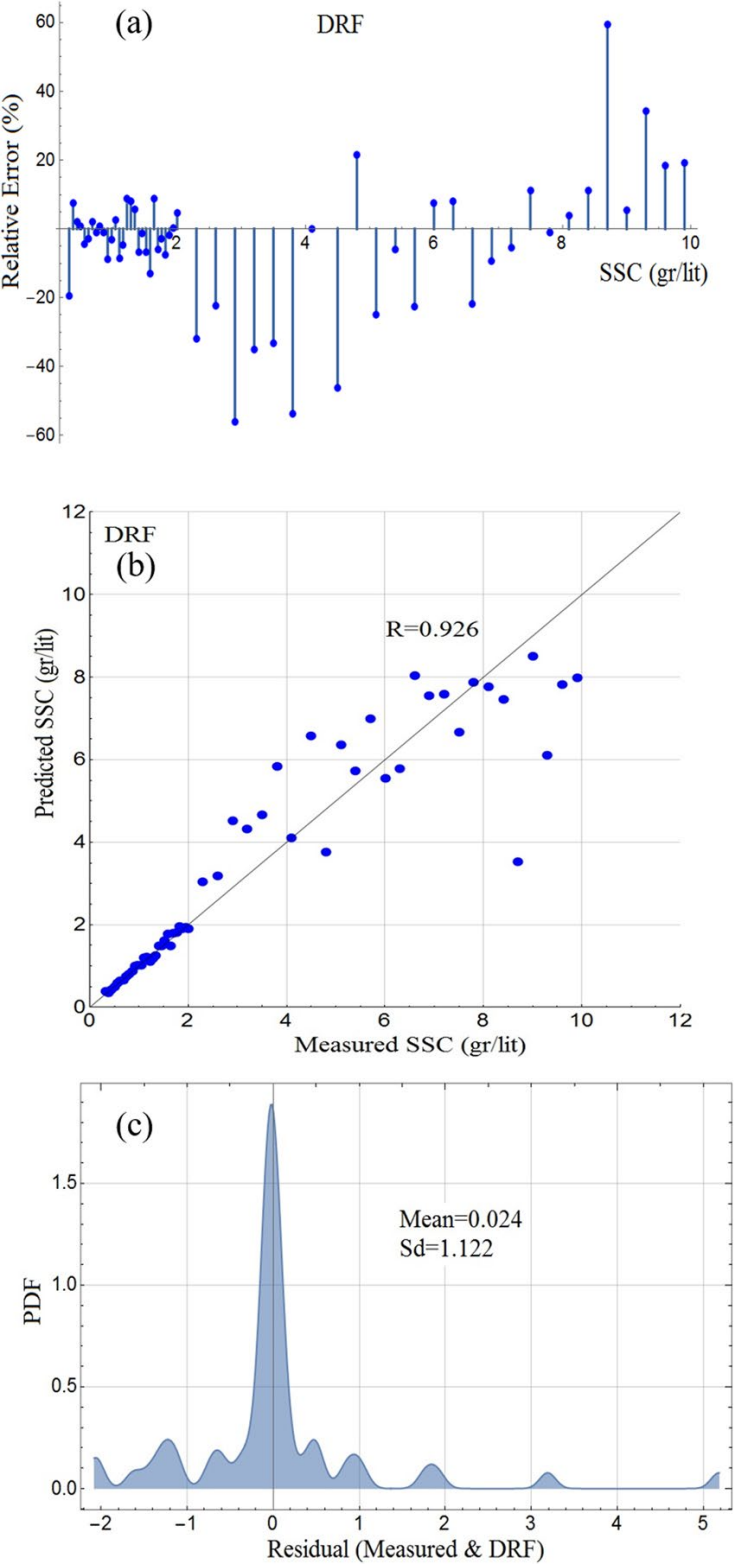
Results of DRF for the testing phase: (**a**) Rlative error plot; (**b**) scatter diagram; (**c**) PDF plot of residuals.

### Model results of generalized linear machine – GLM

The input data to GLM are the image analytics and target values, where the latter are the measured SSC values. The performance of GLM at its testing phase is shown in Fig. 2, in which Fig. 2a shows that Relative Errors (RE) percentages for the model between −5% and 5%, the value of which is approx. 18%; Fig. 2b shows the results for the scatter diagram (modelled SSC against corresponding measured SSC), according to which a large number of datapoints display a good correlation but there are some strong discordant predictions at higher ranges; Fig. 2c displays Probability Distribution Function (PDF) of the residuals and its statistics (mean and standard deviation); according to which the PDF distribution is not similar to the normal distribution and is not symmetric with respect to the centreline; but these are attributable to possible gross errors at higher ranges. Therefore, GLM may be considered as fit-for-purpose for most of the concentration values but at higher values the algorithm can be responsible for undue errors, as discussed later.

**Figure 4:**
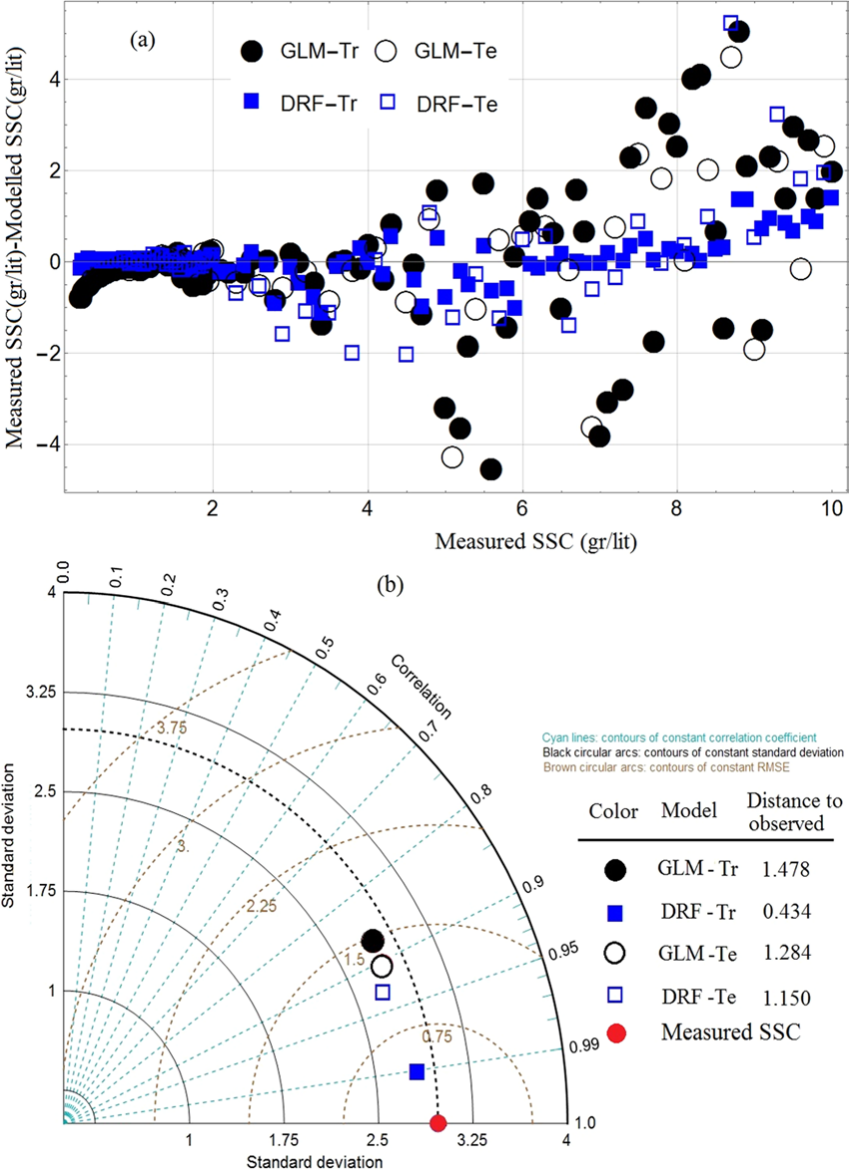
Inter-comparison of the r esults - testing and training phases: (**a**) Scatter diagram of residuals of the models; (**b**) Taylor diagram.

### Model results of distributed random forest – DRF

The input data to DRF are the image analytics and the measured target SSC values. The performance of DRF at the testing phases is shown in Fig. 3a–c.

**Figure 5:**
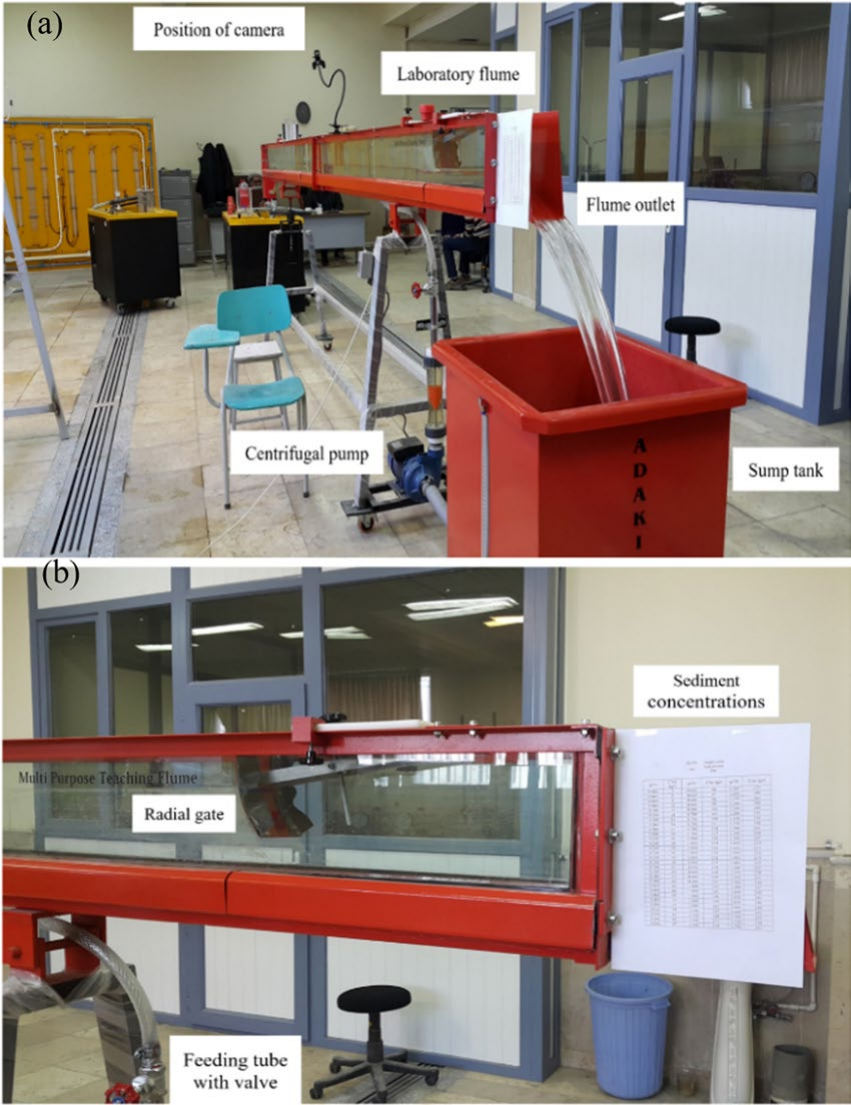
Laboratory setup. (**a**) Experimental setup at the laboratory of hydraulics in the University of Tabriz and the Islamic Azad University of Tabriz, (**b**) downstream of the flume.

The results at the testing phase in Fig. 3a shows that RE percentages for the model between −5% and 5% are approximately 34%; Fig. 3b shows the scatter diagram, according to which a large number of datapoints display a good correlation at low and medium ranges but strong discordant predictions are also observed at higher ranges; and Fig. 3c displays the PDF of the error residuals and its statistics (mean and standard deviation), according to which, the PDF distribution does not quite follow the normal distribution; and have multiple tails. Notably, discordant datapoints at higher values are not many.

### Inter-comparison of models

The goal of the paper is not to search for the best model to predict SSC values from image analytics but to explore their predictability from the analytics. The two ML models are only a means to an end but not the end (the goal). Therefore, no ranking is intended to be carried out by the inter-comparison of these two models. Performances of both models in Fig. 4a are shown in terms of the scatter diagram of their residuals (measured SSCs-modelled SSCs) for both training and testing phases, which provide visual evidence that GLM and DRF are fit-for-purpose for prediction but may suffer from excessive errors at larger SSC values. Hence, their predictions may not be quite defensible and further improvements are appropriate.

**Figure 6:**
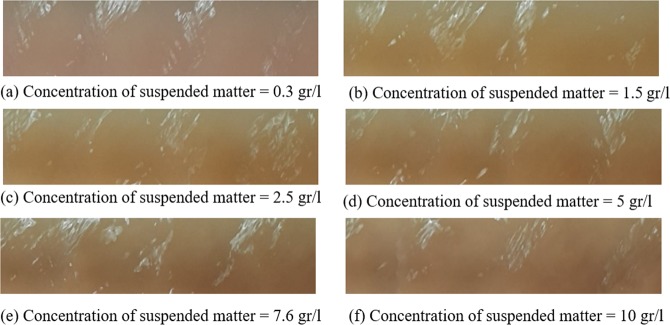
Sample images of water flowing in the flume with different sediment concentrations of (**a**) 0.3, (**b**) 1.5, (**c**) 2.5, (**d**) 5, (**e**) 7.6 and (**f**) 10 gr/l (flow direction is from left to right).

Further comparisons are presented in Fig. 4b, in which the Taylor diagram shows the performance metric for both training and testing phases using SD and RMSE and compares modelled results with observed (measured) values (a single point in red). Thus, the closer the position of the modelled values to that of the observed value, the better the performance. According to Fig. 4b, the position of the DRF results is slightly closer to that of the observed value at both training and testing phases than those of GLM results but significant deterioration is observed at the testing phase. The results provide evidence that there is a significant correlation between image analytics and their corresponding observed SSC values, although attention should be given to significant errors at higher SSC values.

## Discussion and further works

Impacts of Suspended Sediment Concentration (SSC) on water quality is a driver to develop a continuous monitoring capability to minimise turbidity for encouraging subsequent penetration of sunlight to aquatic photosynthesis of algae and other aquatic plants in waterbodies or open channels. The paper provides some evidence for a solution at its proof-of-concept stage, which refers to one stage at the lifecycle of transforming an idea towards the delivery of working tools at 9 steps, as formalised through a procedure given by NASA^18^. The status of the evidence produced by the paper for a continuous SSC monitoring capability should be equivalent to Technical Readiness Level 3 (TRL3) and this would justify future works towards delivering guidelines at TRL9.

The paper presents no comparison with published results, as to the best of the authors’ knowledge, similar research works are yet to be published. Past studies using microscopic image processing have been focussed on studying flocculation processes, e.g. Shen and Maa^19^ and Klassen *et al*.^20^ and such studies are often costly. However, the work by Ramalingam and Chandra^21^ is closer to the present research for investigating suspended sediments but they study their deposition to predict particle size distribution through their settling velocity by an image capturing system, which uses low-cost digital cameras. Their study produces satisfactory results for data obtained from both laboratory and field studies. Notably, the limitation of using images for continuous SSC monitoring is that it is unable to measure vertical distributions but this is currently investigated by holographic techniques, e.g. Graham and Smith^22^.

Before producing guidelines, the paper identified some problems for predicting at higher SSC values that need to be solved. The authors attribute this to possible shortfalls in achieving steady state flows at higher concentration, which can be investigated by attention to the following aspect: (i) using larger flumes with higher capacities; (ii) standardising the laboratory procedure to ensure that suspended matters are well mixed and steady state is ensured; (iii) testing the performance of different suspended matter; (iv) piloting the emerging knowhow on prototype river systems; and (v) developing practice guidelines.

It is noted that no detailed statistical analysis is carried out to establish the probability distribution of the PDFs presented in Figs. 2c and 3c. Arguably, such analyses ensure that the models extract maximum information from the data. However, the authors think that some of the measured data need to be improved and therefore any further statistical analysis is not going to compensate for possible measurement errors.

## Conclusion

The paper presents evidence for the proof-of-concept for a continuous monitoring capability of Suspended Sediment Concentration (SSC) in channels/rivers. It tests the transformation of high-resolution images of the flows carrying suspended sediments through a laboratory flume into image analytics to serve as input data into machine learning models. The models explored inherent correlations between measured SSC and image analytics, comprising 8 input variables: Mean_3_, Mean intensity, Entropy, and Standard deviation_3_ (the suffix ‘3’ corresponds to images with Red, Green and Blue colours).

Although the paper uses machine learning techniques, models at this stage are treated as a means to an end and there is no effort in the paper to search for more appropriate modelling strategies. The two machine learning models comprise: Generalized Linear Machine (GLM) and Distributed Random Forest (DRF). The dataset comprises 166 datapoints, divided randomly into 111 training datapoints and 55 testing datapoints (a ratio of 2/3 to 1/3). The modelling results show that the use of high-resolution image is appropriate for predicting the SSC values. The paper offers evidence for existence of correlation between subsequent image analytics and SSC values and shows the correlation to be strong enough. Thus, the procedure investigated here treats a technological gap and offers a potential for the capability to monitor continuously SSC values. Nonetheless, significant errors are not ruled out at higher concentrations, which are attributed to laboratory procedures and the paper recommends standardisation of the testing procedure, after which prototype pilot studies can be carried out in real river situations before developing practice guidelines.

## Methods

### Laboratory procedure

Experiments were conducted in the hydraulics laboratory in the University of Tabriz and the Islamic Azad University of Tabriz, Iran. The laboratory flume is 8 m long, 0.1 m wide, and 0.4 m deep (see Fig. 5). At the upstream end, a centrifugal pump is connected to the flume with a maximum capacity of 8.7 litre/second; at the downstream end, the flume is connected to a sump tank with the capacity of 0.4 m^3^. The highest flow through the flume would therefore fill the sump tank in 46 sec. The flow through the flume is controlled by a radial gate at the downstream of the flume. The matter in suspension is introduced by mixing the sediment with water in the sump tank as per specified concentration. The study used sediments with a clay texture to achieve the suspended state. The weight of the required clay soil in each concentration was measured with an accuracy of $\pm 0.01$ gr using a digital scale.

During all tests, the temperature of circulation water was measured and its value was 20 °C. Photographs by camera were taken in the middle of the flume (i.e., 1.0 m above the flume) for each concentration and repeated for all 166 sediment concentration datapoints (Fig. 6), during which steady conditions were achieved to minimise impacts of turbulence by the boundary conditions at the upstream and downstream ends.

Whilst there are barriers for a continuous SSC monitoring for a number of reasons including cost, the paper show that the barrier may be removed by a rather cheap solution, as high-resolution cameras are rather cheap nowadays.

### Specification of modelling techniques

The paper specifies the two models (GLM and DRF) used in this study for predicting SSC using image analytics in the H2O platform as described by Landry *et al*.^23^. Each of these models is widely used and well established and H2O is built on Java, Python and R to optimise machine learning for big data and as such, its advantages include efficient data handling facilities for better prediction. An H2O model can handle billions of data rows in-memory, even with a fairly small cluster. It implements almost all common ML algorithms but data preparation and handling facilities use entirely the R Studio software^24^. The continuous SSC monitoring procedure is schematised in Fig. 1 and both models are specified below.

### Generalised linear models – GLM

GLMs connect multivariable inputs to outputs in their predictor mode for regression analysis. Francke *et al*.^25^ investigated the performance of GLM and a set of other models (RF, and Quantile Regression Forest) using data from a flood season at four catchments with different sizes in the Central Spanish Pyrenees. Cox *et al*.^26^ used GLM to estimate SSC in case studies at Burnhope Burn. Its implementation in the paper follows that described by Nykodym *et al*.^27^ and is specified as follows.

The H2O implementation extends the widely-used linear regression analysis as a machine learning technique by maximising the log-likelihood, where the innovation over traditional capabilities is by removing the requirement for the normality of the error distributions through adding an explicit error term as a function of mean, non-normal errors, and a non-linear relation between the response and covariates. Therefore, the response distribution is taken in terms of the exponential family (e.g. the Gaussian, Poisson, binomial, multinomial, and gamma distributions). In this research, the gamma family was selected, which involved a set of default parameters, as specified in Table 2. The estimator mode of the GLM model constructed in this study uses the Iteratively Reweighted Least Squares Method (IRLSM), which applies the Gram matrix approach as the Hermitian matrix of inner products^28^.

### Distributed random forest – DRF

DRF connects multivariable inputs to outputs in their predictor mode, which generates a forest of regression trees, rather than a single regression tree, each of which is a weak learner. Its past applications are wide but to the best of our knowledge it has not yet been applied to investigate SSC. Regression takes on the average prediction over all of their trees to make a final prediction, as more trees reduce variance. DRF is an ensemble of the decision forest algorithms in terms of bagging^29^ and random subspace. In the regression context, Breiman^30^ recommends setting the mean of the tree to be one-third of the number of predictors. For regression models, the prediction error is returned as a mean squared error (MSE). The four tuning parameters used by DRF are specified in Table 2.

### Preparation of data

The primary data comprise experimental images from water flowing through the laboratory flume and their corresponding measured SSC. These are transferred into the Mathematica software to use the function: ImageMeasurements, which calculates their mean, mean intensity, entropy, and standard deviation for each image.

In colour images, measures like mean are given per channel (Red, Green, Blue) and therefore have 3 values for an image. The value of a pixel is its intensity, which refers to the amount of light with reference to a global measure of that image, e.g. means pixel intensity. A relative measure of image intensity can express the brightness (mean pixel intensity) of one image compared with another image. Entropy is a measure of image information content, which is interpreted as the average uncertainty of information source. It is used in quantitative analyses, which provides better comparison of the image details. Standard deviation (SD) gives the deviation of pixel intensity. These values together form 8 variables and are derived from images in the form of image analytics to serve as input data to the models for predicting SSC.

The experimental data, described above, comprise 166 observations, which and their basic statistics is given in Table 1. The two models of GLM and DRF were constructed in the H2O platform through training and testing phases using the input data extracted from image analytics, as outlined in Fig. 1. The default parameters of each of these models are given in Table 2.

### Performance metrics

The following metrics are used to evaluate the performance of the two models: (i) Root Mean Squared Error (RMSE), which shows the discrepancy between observed and predicted values. A value of zero reflects a ‘perfect’ prediction. The lower the RMSE value, the better the model performance. (ii) Correlation Coefficient (CC) shows the correlation structure between observed and modelled values and the higher its value, the greater the correlation and the lesser the deviation. (iii) Relative Error (RE) is the ratio of the absolute error (modelled value minus measured value) by the modelled value and provides an indication of how good a measurement is relative to the size of the measured variable, which is a good reflection of the maximum error. The paper also used the Taylor diagram^30^, which provided a visual representation of observed and modelled data through a single diagram to summarise multiple aspects of observed and modelled values incorporating RMSE and CC.

### Ethical approval

This article does not contain any studies with human participants or animals performed by any of the authors.
